# Intralesional versus intracoronary administration of glycoprotein IIb/IIIa inhibitors during percutaneous coronary intervention in patients with acute coronary syndromes: A meta-analysis of randomized controlled trials

**DOI:** 10.1097/MD.0000000000008223

**Published:** 2017-10-27

**Authors:** Binjie Sun, Zhen Liu, Hongshan Yin, Tao Wang, Tao Chen, Sen Yang, Zhian Jiang

**Affiliations:** a Department of Cardiology, The Third Hospital of Hebei Medical University; b Department of Epidemiology and Health Statistics, Center for Disease Control and Prevention of Hebei Province, Shijiazhuang, P.R. China.

**Keywords:** acute coronary syndrome, glycoprotein IIb/IIIa inhibitors, intracoronary administration, intralesional administration, no-reflow, percutaneous coronary intervention

## Abstract

**Background:**

Glycoprotein IIb/IIIa inhibitors (GPIs) have been regarded as an adjuvant regimen to deal with no-reflow. However, whether intralesional (IL) administration of GPIs improves myocardial reperfusion without increasing bleeding in patients with acute coronary syndrome (ACS) compared with intracoronary (IC) administration has not been well addressed. Our meta-analysis aimed to evaluate the efficacy and safety of IL versus IC administration of GPIs for patients with ACS during percutaneous coronary intervention.

**Methods:**

We systematically searched Medline, Embase, the Cochrane Central Register of Controlled Trials, and Cambridge Scientific Abstracts from January 2007 to May 2017. Thrombolysis in Myocardial Infarction (TIMI) 3 flow, corrected TIMI frame count (CTFC), and complete ST-segment resolution (>70%) were selected as the primary outcomes. Major adverse cardiac events (MACEs) were the secondary outcome, and major bleeding complications were the safety outcome. Data analysis was conducted using the Review Manager 5.3 software.

**Results:**

Six randomized controlled trials were included in our meta-analysis. Compared with IC, IL obtained better results in terms of TIMI grade 3 flow [odds ratio (OR) 2.29; 95% confidence intervals (CIs) 1.31–4.01; *P* = .004], CTFC [weighted mean difference (WMD) -4.63; 95% CI -8.82 to -0.43; *P* *=* .03], and complete ST-segment resolution (OR 1.55; 95% CI 1.12–2.14; *P* = .008). There was a trend toward decreased MACE in the IL administration groups, which was not of statistical significance (OR 0.63; 95% CI 0.30–1.31; *P* = .22). No significant difference was found between the two groups in terms of in-hospital major bleeding events (OR 2.52; 95% CI .66 to 9.62; *P* = .18).

**Conclusion:**

IL administration yielded favorable outcomes in terms of myocardial tissue reperfusion as evidenced by the improved TIMI flow grade, CTFC, complete ST-segment resolution, and decreased MACE without increasing in-hospital major bleeding events. The IL administration of GPIs can be recommended as the preferred regimen to guard against no-reflow.

## 1 Introduction

Percutaneous coronary intervention (PCI) has become the most effective treatment for acute coronary syndrome (ACS). However, a large proportion of patients present with a persistent impairment of microcirculation, which results in the no-reflow phenomenon, a serious complication leading to poor prognosis.^[1]^

Glycoprotein IIb/IIIa inhibitors (GPIs) have been widely used to guard against no-reflow.^[2]^ Several meta-analyses have demonstrated that intracoronary (IC) administration of GPIs improves clinical outcomes compared with intravenous (IV) administration.^[3–5]^ However, IC administration does not lead to optimal contact between the lesion and the GPIs, which are washed out in a short time by the coronary flow. Whether intralesional (IL) administration, which can achieve a higher local drug concentration, offers a better choice is controversial.

Although some randomized controlled trials (RCTs) have compared IL and IC administration of GPIs, these studies suffered from both limited sample sizes and conflicting outcomes. Consequently, we conducted this meta-analysis to evaluate the efficacy and safety of IL administration of GPIs.

## 2 Methods

### 2.1 Ethical review

No ethical committee approval or patient consent was required for this article, as all analyses were based on previously published studies.

### 2.2 Search strategy

We thoroughly searched Medline, Embase, the Cochrane Central Register of Controlled Trials, and Cambridge Scientific Abstracts for all RCTs on the safety and efficacy of IL versus IC administration of GPIs in the patients with ACS from January 2007 to May 2017. The search terms used included “intracoronary,” “intralesional,” “local delivery,” “glycoprotein IIb/IIIa inhibitors,” “abciximab,” “tirofiban,” “eptifibatide,” “percutaneous coronary intervention,” “randomized controlled trial,” “no-reflow,” “microcatheter,” “infusion catheter,” “aspiration catheter,” “balloon catheter,” “self-made balloon with side hole,” and “ClearwayRX catheter.” In addition, the included studies were manually researched.

### 2.3 Eligibility criteria

A study was considered eligible if it met all of the following criteria: the patients had ACS and underwent PCI; administration of GPIs; IL was compared with IC; reported one of the following outcomes: Thrombolysis in Myocardial Infarction (TIMI) 3 flow, corrected TIMI frame count (CTFC), complete ST-segment resolution (>70%), major adverse cardiac events (MACEs), or bleeding events; was an RCT; discussed the no-reflow phenomenon.

### 2.4 Data extraction and quality assessment

We extracted data from each RCT included in the meta-analysis. The following details were extracted: the first author's name, year of publication, study design, inclusion and exclusion criteria, number of patients, age, gender, disease, drug and intervention protocol, endpoints, and follow-up.

The primary outcomes were TIMI flow grade, CTFC, and complete ST-segment resolution (>70%). The secondary outcome was MACE at 6 to 12 months. MACE was defined as the composite of cardiac death, reinfarction, or target vessel revascularization. The safety outcome was major bleeding complications according to TIMI and Global Utilization of Streptokinase and Tissue Plasminogen Activator for Occluded Coronary Arteries bleeding definitions.

All the data were independently extracted from all eligible studies by 2 reviewers. Any disagreements were resolved by a third investigator. If necessary, we consulted with the author of the original article.

The assessments of study quality were based on the Cochrane Collaboration's tool for assessing the risk of bias. Each item was classified as high risk, low risk, or unclear; high risk for a high risk of bias, low risk for a low risk of bias, and unclear for difficult to decide.

### 2.5 Statistical analysis

Pooled weighted mean differences (WMDs) and 95% confidence intervals (95% CIs) were calculated to estimate continuous variables, and pooled odds ratios (ORs) and 95% CIs for numeric data. The *I*^2^ statistic was used to assess heterogeneity among studies. If substantial heterogeneity was observed (*I*^2^ > 50%), a random-effect model was performed. Otherwise, a fixed effects model was selected. A 2-sided *P* value ≤.05 was considered statistically significant. Sensitivity analyses were performed to investigate the origin of potential heterogeneity by excluding 1 trial at a time, allowing us to evaluate the contribution of each trial to the overall estimate. All analyses were conducted using the Cochrane Collaboration Review Manager Version 5.3 software (The Nordic Cochrane Center, The Cochrane Collaboration, Copenhagen, Denmark).

## 3 Results

### 3.1 Search results and basic information

**Figure 1:**
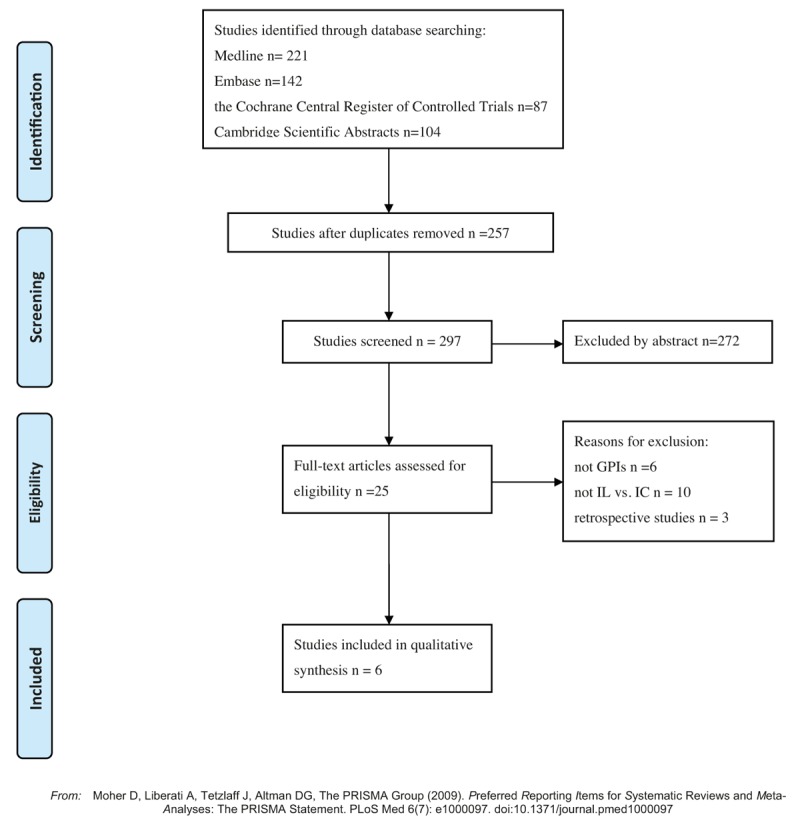
Flow diagram of study selection.

In total, 554 potential studies in Medline (221), Embase (142), the Cochrane Central Register of Controlled Trials (87), and Cambridge Scientific Abstracts (104) were reviewed. A flow diagram of the article selection process is shown in Fig. 1. A total of 6 RCTs involving 751 patients with 386 and 365 receiving IL and IC administration, respectively, were enrolled in our meta-analysis.^[6–11]^ Three RCTs examined abciximab, and 3 tirofiban. Five of the six RCTs enrolled only patients with ST-elevation myocardial infarction (STEMI), while the other RCT enrolled a cohort in which 38% were patients with STEMI. The enrolled studies’ characteristics are presented in Table 1.

**Table 1:**
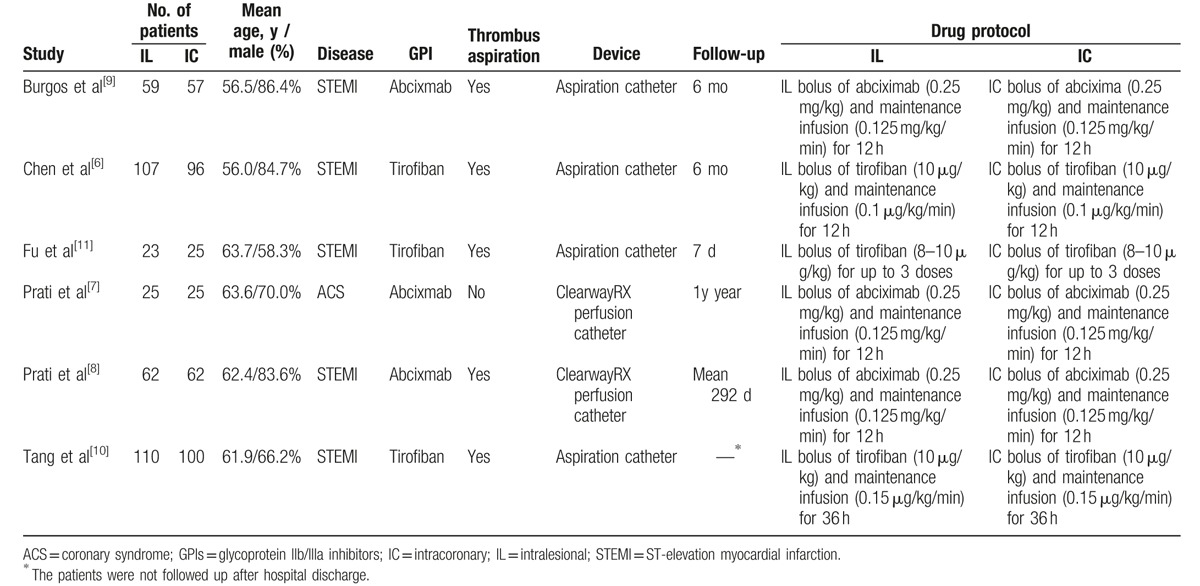
Characteristics of patients and interventions in included studies.

### 3.2 Assessment of study quality

**Figure 2:**
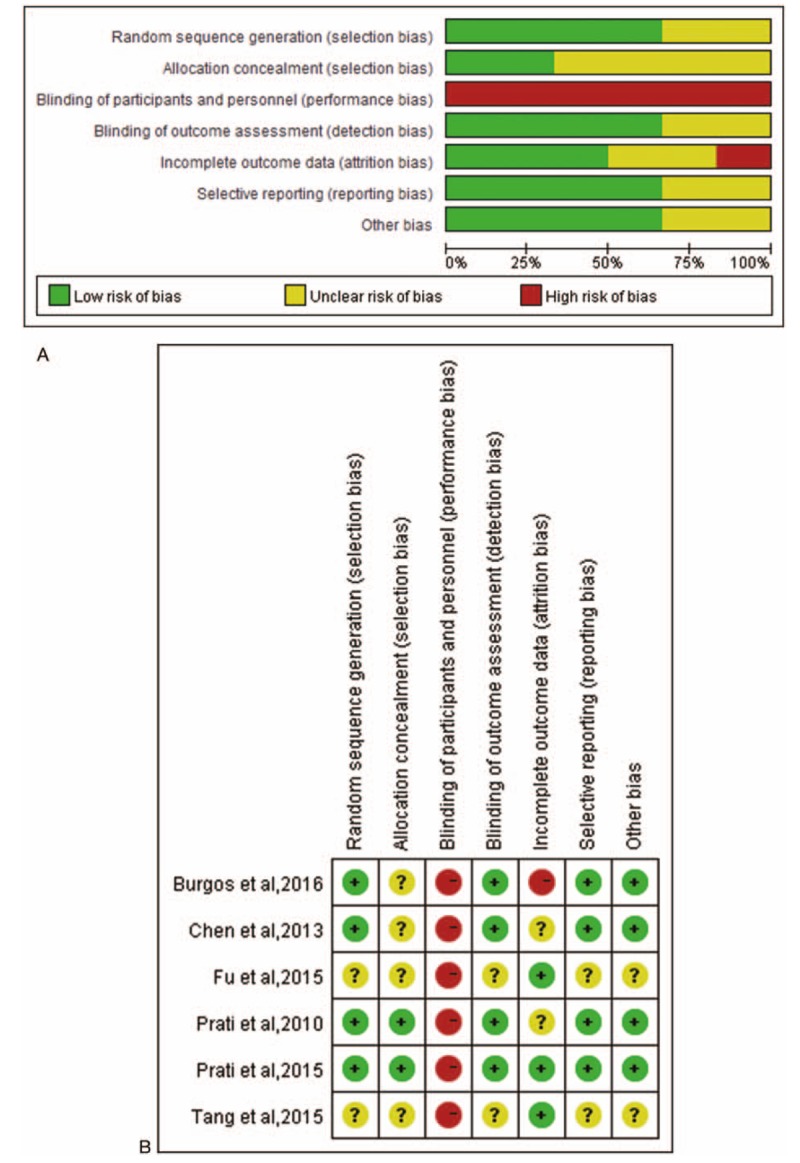
Summary assessments of risk of bias. (A) Risk of bias graph: review authors’ judgments according to each risk of bias item presented as percentages across all included studies. (B) Risk of bias summary: review authors’ judgments according to each risk of bias item for each included study.

The assessment of each RCT's quality is shown in Fig. 2A and B. Given the small number of eligible studies, no study was excluded on the basis of its design characteristics.

### 3.3 Outcomes measures

#### 3.3.1 The primary outcomes

**Figure 3:**
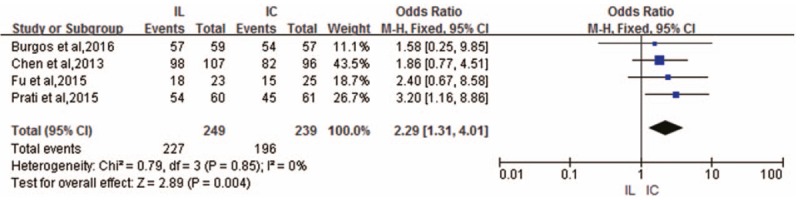
Forest plot of OR for TIMI grade 3 flow.

Four studies reported TIMI flow grade outcomes after PCI.^[6,8,9,11]^ No heterogeneity across these studies was observed (*I*^2^ = 0%). We found that IL administration was more effective in improving the TIMI flow grade (OR 2.29; 95% CI 1.31–4.01; *P* = .004) according to the fixed-effects model (Fig. 3).

**Figure 4:**
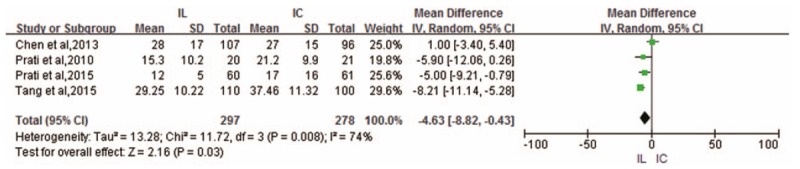
Forest plot of WMD for CTFC.

Four RCTs provided data on CTFC outcomes.^[6–8,10]^ There was significant evidence of heterogeneity (*I*^2^ = 74%) across these RCTs; hence, the fixed-effects model was selected. Compared with IC administration, IL administration proved to be superior in reducing CTFC (WMD -4.63; 95% CI -8.82 to -0.43; *P* = .03) (Fig. 4).

**Figure 5:**
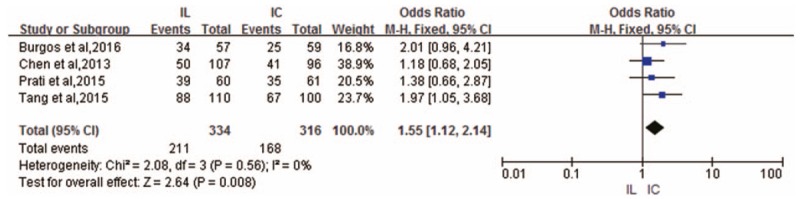
Forest plot of OR for complete ST-segment resolution.

Complete ST-segment resolution (>70%) outcomes were pooled from 4 RCTs.^[6,8–10]^ The incidence of complete ST-segment resolution was higher in the IL administration group than in the IC administration group (OR 1.55; 95% CI 1.12–2.14; *P* = .008) without heterogeneity (*I*^2^ = 0%) across these RCTs (Fig. 5).

#### 3.3.2 The secondary outcome

**Figure 6:**
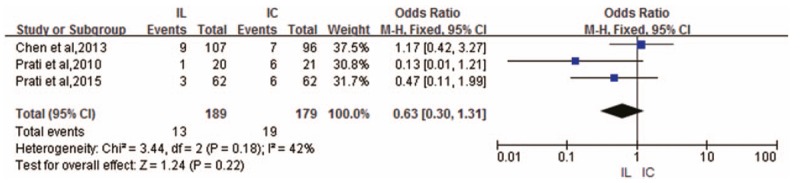
Forest plot of OR for MACE.

MACE outcomes were reported in only 3 RCTs and indicated a trend toward a decrease after IL administration that did not reach significance (OR .63; 95% CI 0.30–1.31; *P* = .22) with a relatively low heterogeneity (*I*^2^ = 42%) across these RCTs ^[6–8]^ (Fig. 6).

#### 3.3.3 The safety outcome

**Figure 7:**
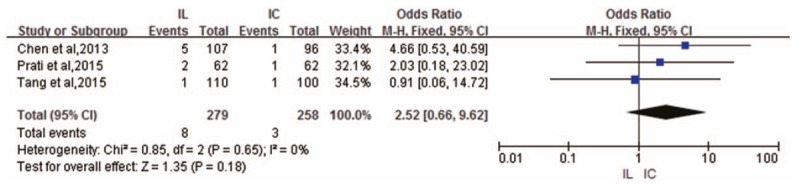
Forest plot of OR for in-hospital bleeding events.

Bleeding outcomes were also provided in only 3 RCTs.^[6,8,10]^ There was no heterogeneity across these RCTs (*I*^2^ = 0%), and no significant differences were observed in terms of in-hospital bleeding events between IL administration and IC administration (OR 2.52; 95% CI 0.66–9.62; *P* = .18) (Fig. 7).

#### 3.3.4 Sensitivity analyses

Sensitivity analysis was conducted for CTFC outcomes with high heterogeneity. The study by Chen et al^[6]^ was found to possibly affect the stability of pooled results. After removing the study, the heterogeneity decreased from high to zero, with the *I*^2^ index decreasing from 74% to 0%, revealing it as the source of heterogeneity. The reason might be ascribed to the higher baseline CTFC of patients in this study.

### 3.4 Publication bias

According to the Cochrane Handbook for Systematic Reviews of Interventions version 5.1.0, publication bias was not determined, on account of the small number of eligible RCTs (<10) in the meta-analysis.

## 4 Discussion

PCI has been regarded as the best available reperfusion strategy in patients with ACS. Despite reestablishing the epicardial coronary vessel patency, PCI may fail to restore myocardial tissue reperfusion. This phenomenon is characterized by no-reflow.^[12–15]^ No-reflow is defined as the inability to reperfuse a myocardial region after prolonged ischemia despite reopening of the infarct-related artery.^[16]^ No-reflow is an independent predictor of prognosis.^[1,17]^ Its underlying pathological mechanisms include distal thromboembolism, injury related to ischemia reperfusion, endothelial dysfunction, diffuse myocardial edema, neutrophilic plugging, and spasms of the microcirculation.^[18,19]^ Okamura et al^[20]^ found that distal thromboembolism resulting from detachment of embolic particles was a common phenomenon that was also the main reason for no-reflow.

It has been reported that many drugs could prevent and treat no-reflow, such as GPIs, adenosine, nicorandil, verapamil, diltiazem, sodium nitroprusside, nitroglycerin, adrenaline, and anisodamine.^[21–24]^ GPIs, the most powerful of antiplatelet agents, play an important role in inhibiting the activation, adhesion, and aggregation of platelets, reducing the release of inflammatory factors, and improving endothelial function. They have shown significant benefits in restoring the antegrade coronary flow of the occluded vessel, preventing and treating no-reflow and reducing the incidence of ischemia events.^[25,26]^

The previous RCTs and meta-analyses have shown that IC administration of GPIs in patients with STEMI yielded more favorable outcomes in terms of postprocedural blood flow restoration and 30-day clinical prognosis and did not increase the risk of bleeding compared with IV administration.^[3–5,27,28]^ The advantages of IC administration may be attributed to its ability to facilitate a higher local concentration. However, IC administration of GPIs usually leads to flow to other areas of the vascular bed or refluxes into the aorta. Meanwhile, the no-reflow blocks delivery to the distal blood vessels. Furthermore, IC administration by guiding catheter may influence heart rate, oppressing the sinoatrial artery when the drug is delivered into the right coronary artery. Similarly, the method may oppress the left circumflex artery, when the drug is delivered to the left anterior descending, which may lead to a drop in blood pressure, hemodynamic instability, severe bradycardia, and may even trigger sinus arrest.

Our meta-analysis demonstrated for the first time that IL administration of GPIs has positive benefits compared with IC administration. IL administration by special catheters (such as aspiration catheters, ClearWayRX perfusion catheters (Atrium Medical Corporation, Hudson, New Hampshire), microcatheters, and self-made side hole balloon catheters) improved myocardial tissue reperfusion, which was reflected by improvements in the TIMI flow grade, CTFC, and complete ST-segment resolution (>70%). Fu et al^[11]^ demonstrated that IL administration could also improve TIMI myocardial perfusion grade. Although the incidence of MACE at 6 to 12 months was not significantly different between the 2 groups, the IL administration groups had a tendency toward lower incidences than the IC administration groups.

There are 2 potential mechanisms contributing to the advantages of IL administration. First, as the drug can be directed toward the target coronary, IL administration can achieve a higher local drug concentration and greater bioavailability. This method can also prolong drug residence time at the site of the thrombus and avoid the loss of the drug down the uninvolved coronary artery or through the aorta. The high concentration can yield superior thrombus disaggregation and less microembolization of the thrombus, which results in better microvascular perfusion.^[29–32]^ Second, according to the principles of physics, IL administration can yield a higher pressure and perfusion flow rate, which would lead to a more pronounced effect at distal lesions.

GPIs may increase the incidence of bleeding events due to their antiplatelet activity and antithrombotic properties. However, there was no significant difference in in-hospital bleeding events between the 2 procedures in our meta-analysis. The outcome is not surprising considering that the same drug at the same total dosage and duration was administered, that caution was exercised during the administration of antiplatelet agents, and that attention was paid to patient management.

## 5 Limitations

Several potential limitations exist in this meta-analysis. First, the occurrence of no-reflow is a complex process involving multiple factors; therefore, its prevention and treatment require individual and combinative strategies. However, owning to the limited number of RCTs and databases, we could not perform subgroup analysis to uncover which patients benefit most from IL administration. In addition, we did not analyze whether other drugs could equally improve myocardial perfusion and evaluate the cost of different strategies. On the basis of the above limitations, more large-scale, high-quality RCTs involving cost-effectiveness analysis need to be designed to further evaluate the merits of IL administration. Second, we included studies on all GPIs regardless of pharmacologic mechanism. Fortunately, a previous mete-analysis had found that there was no significant difference in patients treated with abciximab and the small-molecule GPIs (tirofiban and eptifibatide).^[33]^ Finally, our meta-analysis had inherent limitations, which include publication bias, selection bias, and within-study bias.

## 6 Conclusion

IL administration yielded favorable outcomes in terms of myocardial tissue reperfusion as evidenced by the improved TIMI flow grade, CTFC, complete ST-segment resolution, and decreased MACE without increasing in-hospital major bleeding events. The IL administration of GPIs can be recommended as the preferred regimen to guard against no-reflow.

## Acknowledgment

We thank the patients taking part in the original studies, the authors providing the data, and Editage [www.editage.cn] for English language editing.
